# Supplementary material for: Modeling human intuitions about liquid flow with particle-based simulation
Source: PLoS Comput Biol. 2019 Jul 22;15(7):e1007210. doi: 10.1371/journal.pcbi.1007210 (PMC6675131; doi:10.1371/journal.pcbi.1007210)
Supplement: S1 Appendix — (PDF) [file pcbi.1007210.s001.pdf]

S1 Appendix A: Simulation in Blender. The stimuli in both experiments used Blender's (www.blender.org) lattice-Boltzman liquid simulator. All simulations used a grid resolution of 300 and 100 tracer particles. The water stimuli used Blender's preset kinematic viscosity of 1.000 centiPoise. The honey stimuli had a kinematic viscosity of 100.0 cP in Experiment 1 and 2.000 cP in Experiment 2. All other simulation settings were left to their default values (Real World Size: 0.5 meters; Grid Levels: -1; Compressibility: 0.005; Slip Type: Free Slip; Surface Smoothness: 1.000; Surface Subdivisions: 0). Despite being set to free slip, the simulations at high viscosity liquid exhibited adhesive effects. The liquids were rendered using a Mix Shader with 0.05 fraction Diffuse BSDF (Roughness: 0.000) and Beckmann Glass BSDF (Roughness: 0.061, IOR: 1.330).
