# Supplementary material for: Modeling human intuitions about liquid flow with particle-based simulation
Source: PLoS Comput Biol. 2019 Jul 22;15(7):e1007210. doi: 10.1371/journal.pcbi.1007210 (PMC6675131; doi:10.1371/journal.pcbi.1007210)
Supplement: S2 Appendix — (PDF) [file pcbi.1007210.s002.pdf]

S2 Appendix B: Individual Subject Analyses. Figure 6 (main text) revealed that in some cases, subject correlations were not normally distributed. We therefore carried out additional individual subject analyses to investigate whether our averaged-response analyses obscured any important individual-level effects. For example, it could be the case that subjects who had low correlations with ground truth in Exp. 2 water trials were well-explained by a less complex model, such as SimpleSim. To gain insight into these kinds of questions, we redid Figure 6 (main text) for each model in turn, in place of ground truth (see figures A, B, C, and D). That is, we examined the distributions of how individuals correlated with each model (IFE, SimpleSim, MarbleSim, and ConvNet). To summarize, we found that these additional analyses did not yield any further insights. Of note, subject responses in Exp. 2 water trials were similar to the null hypothesis, suggesting that if some subjects used a qualitatively different strategy in Exp. 2 water trials, they were not well-explained by SimpleSim.

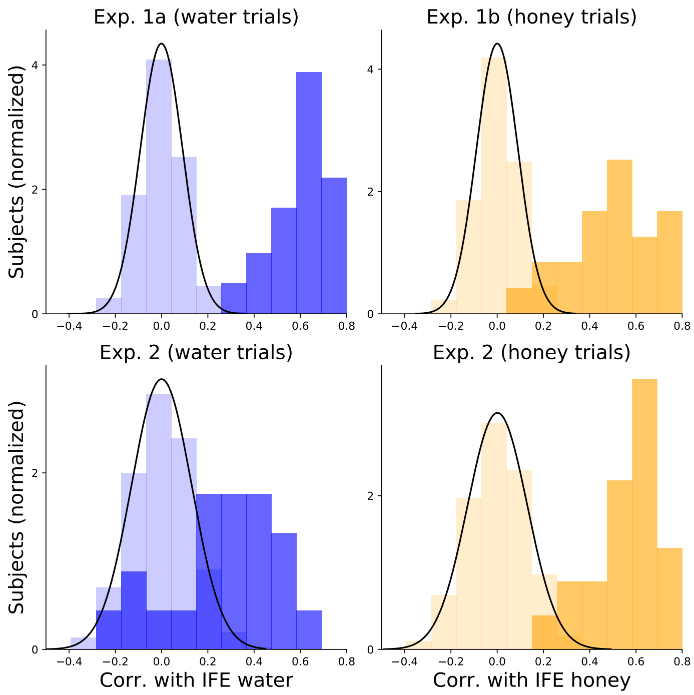

Fig A. Distribution of individual participant correlations with IFE (dark colors) versus the null hypothesis (light colors).

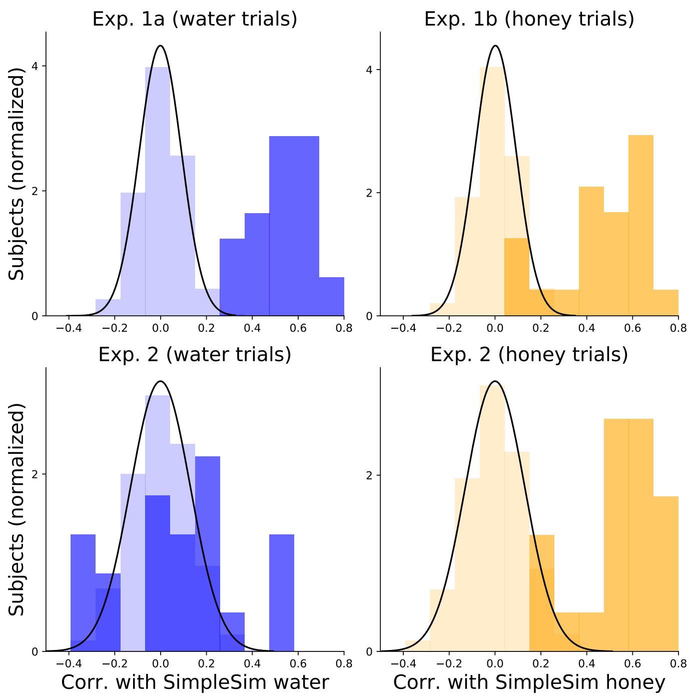

Fig B. Distribution of individual participant correlations with SimpleSim (dark colors) versus the null hypothesis (light colors).

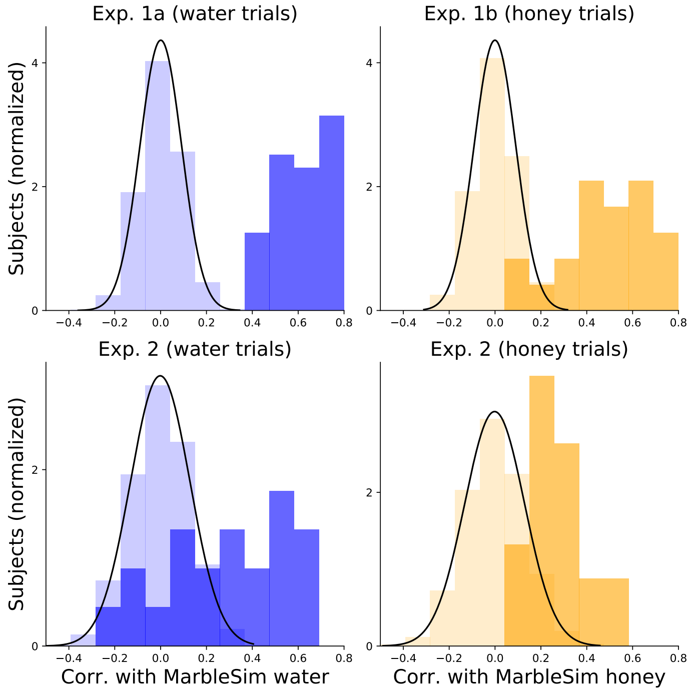

Fig C. Distribution of individual participant correlations with MarbleSim (dark colors) versus the null hypothesis (light colors).

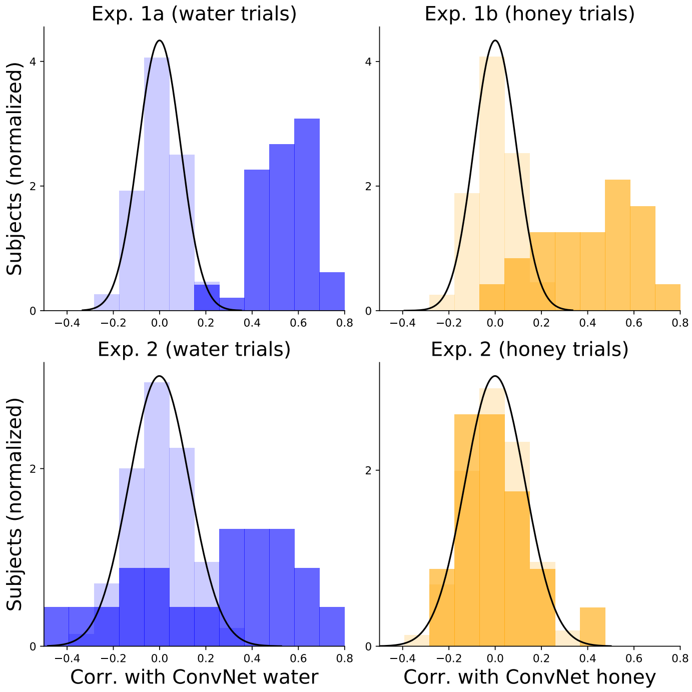

Fig D. Distribution of individual participant correlations with ConvNet (dark colors) versus the null hypothesis (light colors).
